# Massive stone or is it glass: a curious case of porcelain gallbladder

**DOI:** 10.1093/jscr/rjad533

**Published:** 2023-09-24

**Authors:** Tan Jun Guang Kendric, Ruwan Wijesuriya

**Affiliations:** General Surgery, St John of God Midland Hospital, 1 Clayton St, Midland, WA 6056, Australia; General Surgery, St John of God Midland Hospital, 1 Clayton St, Midland, WA 6056, Australia

**Keywords:** porcelain gallbladder, computed tomography, gallbladder malignancy

## Abstract

Usage of computed tomography (CT) scans has increased exponentially over the past decade. This is associated with the rise in incidental findings and having to manage clinical scenarios previously never encountered in the pre-CT scan era. Once such finding is a porcelain gallbladder, specifically gallbladder wall calcification. We report one such case of a porcelain gallbladder mimic and propose some suggestions on the decision-making process when managing an incidentally discovered calcified gallbladder.

## Introduction

Porcelain gallbladder is an uncommon condition where the inner wall of the gallbladder becomes calcified and hardened, taking on a bluish hue and becoming increasingly brittle, hence the ‘porcelain’ description [1]. It has an incidence rate of 0.06% to 0.8% and risk factors include the female gender, increased age, and cholelithiasis. Approximately 90–95% of porcelain gallbladder cases are associated with gallstones [2, 3]. In our case report, we present one such case of an extremely convincing porcelain gallbladder incidentally detected on computed tomography (CT), which surprisingly turned out to be a large rim-calcified gallstone on histology.

## Case presentation

Our patient is a female in her 60s who was referred by her general practitioner after a CT scan of her lumbar spine incidentally revealed peripheral calcification in the gallbladder wall, suspicious for a porcelain gallbladder.

On assessment in our surgical clinic, she is relatively fit and well with a long history of billary colic. She has a past medical history of hypertension, asthma, gastroesophageal reflux disease, open appendectomy, and tubal ligation. Her regular medications include Omeprazole, Escitalopram, vitamin D supplementations, and Budesonide/Formoterol inhaler.

**Figure 1:**
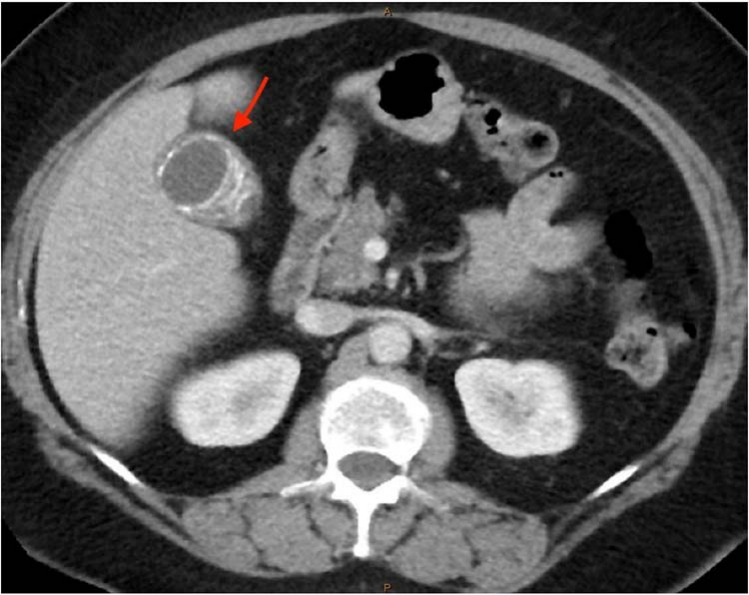
Semi-annular wall calcification at the gallbladder body—axial view.

**Figure 2:**
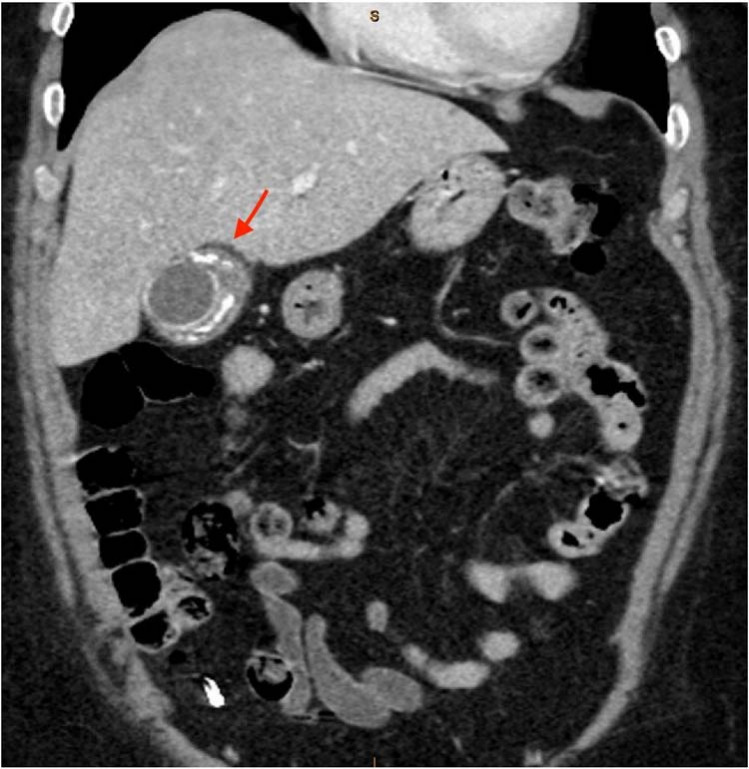
Semi-annular wall calcification at the gallbladder body—coronal view.

**Figure 3:**
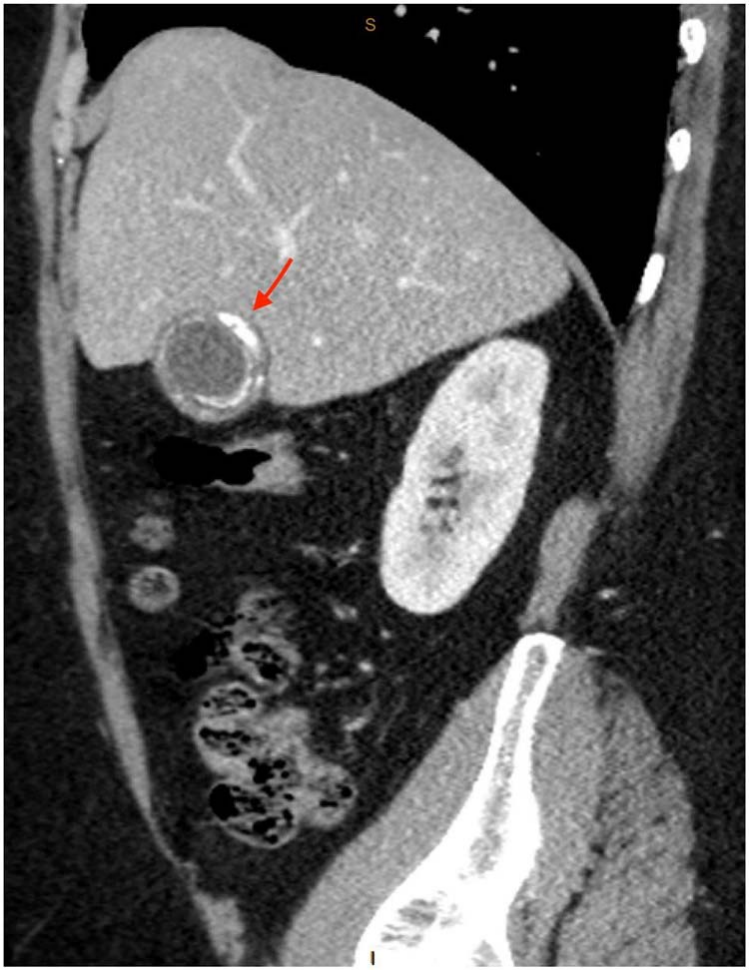
Semi-annular wall calcification at the gallbladder body—sagittal view.

As only part of her gallbladder was imaged on the non-contrast CT lumbar spine, a repeat CT scan of her abdomen and pelvis with portal venous contrast was ordered. This showed semi-annular wall calcification at the gallbladder body suggestive for porcelain gallbladder and several calcified gallstones with largest measuring up to 32 mm (Figs 1–3). No gallbladder soft tissue mass was seen.

Given our patient had minimal medical comorbidities and long-standing billary colic, we decided a cholecystectomy will treat her symptoms as well as remove the risks of malignancy. We proceeded to perform a laparoscopic cholecystectomy and her gallbladder appeared normal externally. Intra-operative cholangiogram was not done to prevent bile leak.

Her initial histopathology report is as follows. Macroscopy showed an intact gallbladder 85 × 40 mm, with smooth serosal surface. The walls are 4 mm. There are two large brown and black stones in aggregate 60 × 45 mm. Microscopy showed chronic cholecystitis with without wall calcification.

Given the strong clinical and radiological suspicion of a porcelain gallbladder, and that small infiltrating malignant single cells or cords may not be appreciated within the dense fibrous and calcified tissue, we requested for a second histopathological review. This again showed no areas of calcification, dysplasia, or malignancy.

In retrospect, we suspect the rim calcification initially visualized on CT scan could have been calcification along the outer rim of the large 60 × 45 mm gallstone, instead of true gallbladder wall calcification.

Our patient was seen in our surgical clinic 1 month postsurgery and counselled on the above findings. She had an unremarkable recovery and given the benign findings, discharged from our service.

## Discussion

Our case highlights a few key points. Surgeons have to appreciate the malignant potential in a porcelain gallbladder and weigh this against the operative risk and clinical benefit, keeping in mind it may be mimicked by calcified gallstones or sludge.

Porcelain gallbladder is thought to be the result of chronic inflammation. The combination of inflammation, dystrophic calcification, errors in calcium metabolism, and ischemia stimulates calcification in the gallbladder wall. An alternate explanation is that chronic obstruction of cystic duct leads to precipitation of calcium salts in the gallbladder’s mucosa layer. Selective mucosal calcification involves focal plaques of gallbladder wall calcification in the mucosal layer. Complete intramural calcification involves the full thickness of gallbladder wall, replacing the muscular layer entirely. There is dense fibrosis of the entire gallbladder wall and sloughing of the mucosal epithelium [1]. Dysplasia and malignancy can be missed in this dense fibrosis, hence the need for meticulous histopathological examination. A systematic literature search in 2012 by T. Schnelldorfer revealed the overall rate of gallbladder malignancy in patients with gallbladder wall calcification was 6% compared with 1% in matched patients without gallbladder wall calcification. This is much lower than the historically quoted 7%–61% chance of malignancy, albeit not an insignificant risk. More importantly, it demonstrated that patients with gallbladder wall calcifications are indeed at a statistically higher risk for gallbladder malignancy [2]. Gallbladder malignancies are largely asymptomatic until the late stages when they are usually diagnosed. This contributes to the poor average 5-year survival rates of 19% [4]. Hence, in light of the 5% increased risk of malignancy in porcelain gallbladders, there is argument for prophylactic cholecystectomy in all patients.

The ideal imaging modality for porcelain gallbladder is CT. Findings are pathognomonic with calcification within gallbladder wall, showing up as curvilinear or rim calcification. However, porcelain gallbladder is frequently overcalled on CT imaging, with nearly one-third of patients having either calcified gallstones or sludge [5]. As CT Imaging increases worldwide for a variety of clinical indications, incidental findings such as porcelain gallbladder are on the rise. Clinicians need to understand the clinical significance, appreciate historical implications but be updated on current literature to make informed decisions for patients.

Given the 6% lifetime risk of gallbladder malignancy, it is clear that offering cholecystectomy to symptomatic patients who are otherwise well is beneficial. However, we believe the role of cholecystectomy in asymptomatic patients with incidental gallbladder calcification depends on patient factors. First, we are inclined to operate on younger patients who have a higher lifetime exposure to the relative risk of gallbladder malignancy [2]. Next, we know that risks of major complications in uncomplicated cholecystectomy are around 3%–4%, with common bile duct injury and peri-operative mortality both at 0.5% [6–9]. In a medically co-morbid patient, if the perceived risks of cholecystectomy are higher than 6%, prophylactic cholecystectomy is not recommended. However, if the perceived operative risks are <6%, the clinician and patient should discuss the risks versus benefits before arriving at a shared decision.

In conclusion, it is reasonable for symptomatic patients or asymptomatic patients who are young and fit to undergo prophylactic cholecystectomy once a porcelain gallbladder is detected. For medically co-morbid patients, it is reasonable to adopt a non-operative approach. However, more research needs to be done to determine the optimal surveillance strategy.

## Data Availability

No new data were generated or analysed in support of this research.

## Patient’s perspective

I am writing a little note of what I have experienced on the matter.

I was not at all aware that I had gallbladder trouble until I went to my local doctor to ask for some facet joint injections as my back was playing up. She looked up my history and said although I had some of these injections over the years, I had not had a scan for 12 years so off I went to get it done. Within a day or so, the doctor rang me and I naturally thought it was about my back. However, she told me if I could come and see her. She explained my gallbladder had shown up on the scan looked calcified and I was then sent to a specialist at St John of God Midland Hospital.

Everything was explained to me and naturally my question was: do I have to have it out being that I am not suffering? I was told that being calcified, there is a good chance that it is or could become cancerous. I was placed on the category 2 waiting list and I heard within 6 months. In the following year, I went into hospital very very nervous as I am not the bravest, but I knew it had to come out. I was treated very well and had the procedure explained to me. I was informed if the operation became difficult, it would not be done through the naval. I woke up feeling lightheaded but soon came round. I was told they did have difficulty removing it but managed to do it through the naval. The gallbladder was tested twice and I was told the scan and histology did not add up, hence the porcelain gallbladder mimic. I recovered very well and was looked after well in hospital. I did return a week later as I managed to get a bad infection at the naval site. The specialist who performed the operation opened my wound and cleaned it all up, and I spent 3 days in hospital with antibiotics.

I have since had no problems whatsoever except watching what I eat as my tummy does not like some things.
